# Combining Deep Learning and Multi-Source GIS Methods to Analyze Urban and Greening Changes

**DOI:** 10.3390/s23083805

**Published:** 2023-04-07

**Authors:** Mauro Francini, Carolina Salvo, Alessandro Vitale

**Affiliations:** Department of Civil Engineering, University of Calabria, 87036 Rende, CS, Italy

**Keywords:** urban development, greening development, remote sensing, deep learning, U-Net, geographic information system, spatial analysis

## Abstract

Although many authors have observed a degradation in greening cover alongside an increase in the built-up areas, resulting in a deterioration of the essential environmental services for the well-being of ecosystems and society, few studies have measured how greening developed in its full spatiotemporal configuration with urban development using innovative remote sensing (RS) technologies. Focusing on this issue, the authors propose an innovative methodology for the analysis of the urban and greening changes over time by integrating deep learning (DL) technologies to classify and segment the built-up area and the vegetation cover from satellite and aerial images and geographic information system (GIS) techniques. The core of the methodology is a trained and validated U-Net model, which was tested on an urban area in the municipality of Matera (Italy), analyzing the urban and greening changes from 2000 to 2020. The results demonstrate a very good level of accuracy of the U-Net model, a remarkable increment in the built-up area density (8.28%) and a decline in the vegetation cover density (5.13%). The obtained results demonstrate how the proposed method can be used to rapidly and accurately identify useful information about urban and greening spatiotemporal development using innovative RS technologies supporting sustainable development processes.

## 1. Introduction

Over the past few years, in the urban planning context, there have been increasing considerations regarding how the combination between urban development and the achievement of urban greening goals can contribute to “make cities and human settlements inclusive, safe, resilient and sustainable” [1]. Many researchers have studied the tension between these two development ambitions. Most have found that urban development—both compact and dispersed [2,3,4]—determines a reduction in the vegetation cover [5,6,7]. It seems, therefore, difficult to increase urban development without losing natural areas.

Urban development should go hand in hand with an increase in vegetation density and distribution in order to prevent the degradation of viable ecosystem services that are critically important for human well-being and livability, such as outdoor recreation, air purification, biodiversity, cooling, carbon storage, water infiltration, noise reduction and air temperature regulation [8,9,10]. Despite this, many authors have observed the degradation of urban greening and an increase in population and built-up density in urban areas, causing a drastic reduction in vegetation cover and, therefore, in ecosystem services provisioning [5,6,7,11].

This complex relationship between urban and greening development goals is addressed across the literature [5,6,7]. However, few studies have measured how greening has developed in terms of its full spatial-temporal configuration with urban development, especially using innovative remote sensing (RS) technologies, which can be very useful for obtaining accurate information while drastically reducing times and costs for those processes. Ref. [12] performed a GIS analysis using RS to investigate land-use changes in Brussels and Amsterdam from 2003 to 2016, focusing on trends in quantity, distribution and the forms of green spaces The RS analysis specifically involved a process of supervised land-use classification. Ref. [13] performed a regression-based unmixing approach on Landsat satellite imagery between 1988 and 2018 to generate fraction maps of vegetated and non-vegetated surfaces.

Urban and greening survey data are not commonly updated or freely accessible to local users. Generally, urban and greening development can be assessed by retrieving the built-up and vegetation cover data from the land use and land cover (LULC) maps. At the European level, one of the most used LULC maps is the Urban Atlas [14]. Additional information can be retrieved, for example, from OpenStreetMap (OSM) or from maps and geo-databases provided by local authorities and public administrations. However, detailed information about the buildings and the vegetation is not always provided, since detailed LULC maps often do not exist and, if available, they are not free of charge. Other traditional methods to obtain urban shapes and vegetation polygons are based on the visual interpretation of aerial imagery and manual digitalization [15]. These methods, requiring a large number of manual operations, are time-consuming and costly. 

RS technologies and their development are very useful for identifying these land elements rapidly as well as analyzing the spatial-temporal trends and changes in urban and greening development. Their application in this field can help urban planners, decision-makers and scientists to assess the current status of urban and greening development and its evolution in space and in time. Based on this information, urban planners and decision-makers can assess the effects of urban planning policies as well as predict future urban and greening development. However, contributions to the urban and greening long time series analysis in urban areas using the new and innovative RS technologies are still rare. 

The knowledge and mapping of land elements using aerial imaging allow the management of the landscape transformation [16] and represent an essential aspect of a wide range of applications, such as urban and regional planning [17], environmental vulnerability [18], natural disasters and hazards monitoring and the estimation of soil erosion [19]. Recently, in the most novel approaches, the application of machine learning (ML) algorithms to RS imagery for land mapping has attracted considerable attention [20,21]. 

ML algorithms can recognize, detect, plan, predict and classify land features using the RS imagery data. ML algorithms can be divided into two different categories: classical/traditional and deep learning (DL) algorithms [22]. Approaches based on classical/traditional algorithms include support vector machine (SVM), random forest (RF), spectral angle mapper (SAM), fuzzy adaptive resonance theory-supervised predictive mapping (Fuzzy ARTMAP), Mahalanobis distance (MD), radial basis function (RBF), decision tree (DT), multilayer perception (MLP), naive Bayes (NB), maximum likelihood classifier (MLC) and fuzzy logic [23,24]. Other techniques include the affinity propagation (AP) algorithm, fuzzy c-means algorithms, K-means algorithm, and iterative self-organizing data (ISODATA) [20,21]. According to the conducted literature review, SVM and RF generally provide better accuracy compared to the other traditional classifier techniques [25,26].

The extraction process of land features from aerial images necessitates a finer-resolution image pixel and a reduced per-pixel size. With the increase in aerial image spatial resolution, the land features have become more clearly visible, and therefore, many studies recently changed approaches, changing from pixel-based image analysis (PBIA) [27] and object-based image analysis (OBIA) [28,29] to pixel-level semantic segmentation. 

In PBIA methods, the pixel size characteristics are not compatible with the identification of an object in an image. In fact, according to [30], for example, a four meter-wide object needs a minimum of four pixels. Furthermore, PBIA methods often generate “salt and pepper” noise after classification. To overcome these disadvantages, OBIA methods were introduced. This different approach identifies a feature as a group of contiguous homogeneous pixels with similar texture, spatial and/or spectral attributes. In recent years, thanks to the evolution of DL-based semantic segmentation, the LULC studies’ accuracy increased. Semantic segmentation can represent the process of assigning a semantic label to each coherent region of an image. This coherent region is represented by a pixel [31], a subpixel [32], a super-pixel [33] or an image patch composed of several pixels [34].

The most recent DL approach represents an advancement of the classical neural networks and includes classifiers such as convolutional neural networks (CNNs) [35,36,37,38,39], recurrent neural networks (RNNs) [40] and deep neural networks (DNNs) [41]. These new algorithms guarantee better results and better RS imagery classification and segmentation in comparison with traditional ML methods. 

The early fully convolutional network (FCN) algorithms were able to identify urban features to a certain extent but had many difficulties because of the loss of high-frequency details, blurred boundaries, and the limited ability to reconstruct spatial information while obtaining rich contextual information [42]. To overcome this limitation, [43] designed the U-Net architecture, adding the multi-layer feature maps from the encoder using the decoder structure for step-by-step upsampling. The U-Net model can be classified as a deep convolutional neural network (DCNN) and thanks to the fusion of high-and-low level semantic information, represents an important improvement to the classification of object boundaries. In fact, DNNs can automatically extract several specific features in RS images to fully realize the classification of urban land use. 

The semantic segmentation of RS images was used to classify and add color to different ground objects in the image and the most applied DCNNs are FCNs, SegNet, U-Net and DeepLab. The U-Net architecture can partially overcome the boundary pixel classification problem in semantic segmentation thanks to its skip connections. A DeepResUnet model was employed by [44] to better perform pixel clustering for building segmentation. The authors used a high-resolution image dataset. Often, the identification of green and built-up areas has to overcome the problem of an unbalanced dataset, which leads to the severe problem of class imbalance in the semantic segmentation of RS images. To limit this inconvenience, several authors used multiple FCNs to form new networks [45], combining the SegNet [46] and U-Net.

In the last few years, different RS image datasets have been created. The labels of each dataset present certain differences [47]. Among others, SEN12MS [48] and the “Semantic Segmentation Dataset” provide pixel-level labels, while BigEarthNet [49] provides image-level labels. In particular, the “Semantic Segmentation Dataset”, an open access dataset developed for a joint project with the Mohammed Bin Rashid Space Center in Dubai, contains 72 labeled satellite images with pixel labels assigned manually. For each pixel, there is, at most, a one-pixel label. All these datasets have many semantic classes, such as buildings, roads, water and land. 

Moreover, in recent years, the combination of the great potential of DL algorithms, capable of classifying and segmenting thousands of RS images, and geographic information systems (GIS), has gained particular importance. Their integration aids in decision making for a variety of applications, including urban and territorial planning and management, sustainable natural resource management and detecting global change issues [50]. GIS and DL can constitute a single source able to provide very important information and enhance strategic decision making using historical data and maps. Ref. [51] calculated the building energy-use intensity at the urban scale using a GIS-integrated data-mining approach, including pre-processing, feature selection and algorithm optimization. Ref. [52] developed a model which combines GIS and DL to estimate the urban land value while considering the variables affecting urban land price and spatial features. All the variables are processed with DL algorithms, creating a deep hybrid neural network with spatial characteristics. Another scientific work presented a combined GIS and CNN model to quantify the influence of location data on property value in Philadelphia [53]. The study highlighted how significant geographical data and high-quality images can improve a model’s accuracy. Ref. [54], using massive street-view datasets, employed DL algorithms for the identification of various urban scenarios in order to quantify the relationship between public perception and urban scenes. 

In this context, the authors of this paper proposed an integrated tool that was able to assess the urban and greening development changes over time through the spatial analysis of the built-up area and vegetation cover classified and segmented from aerial images through the application of a DL model. The proposed method combines DL technologies to classify and segment building heritage and vegetation cover from satellite and aerial images with GIS techniques to import, use and process its output to analyze the urban and greening changes over time. In this study, the authors applied a U-Net model with a RELU activation function that was trained and validated using the “Semantic Segmentation of Aerial Imagery” [55]. The U-Net model was, then, tested with the classification and segmentation of buildings and vegetation cover in an urban area in the municipality of Matera in the Basilicata region (Italy). The output of the model was imported into the GIS environment to investigate the built-up area and the vegetation cover changes in the 2000–2020 period, focusing on their quantity, according to specific urban and greening development indicators. 

The proposed method can help decision makers to identify where and when greening and urban development has grown, as well as the paths, opportunities and threats of these forms of development. The proposed integrated spatial and temporal analysis of urban and greening development can be useful to evaluate and design better urban planning policies. It can be used in different contexts to identify useful information about the urban development, protection and promotion of natural areas rapidly and accurately from satellite and aerial images as well as from unmanned aerial vehicles (UAV) flights.

The rest of the paper is organized as follows. In the next section, the datasets and study area are presented. Section 3 contains the methodological basis of the research, while the results are demonstrated in Section 4. A discussion is presented in Section 5, and Section 6 contains the conclusions of the paper.

## 2. Study Area and Dataset

### 2.1. Study Area

**Figure 1:**
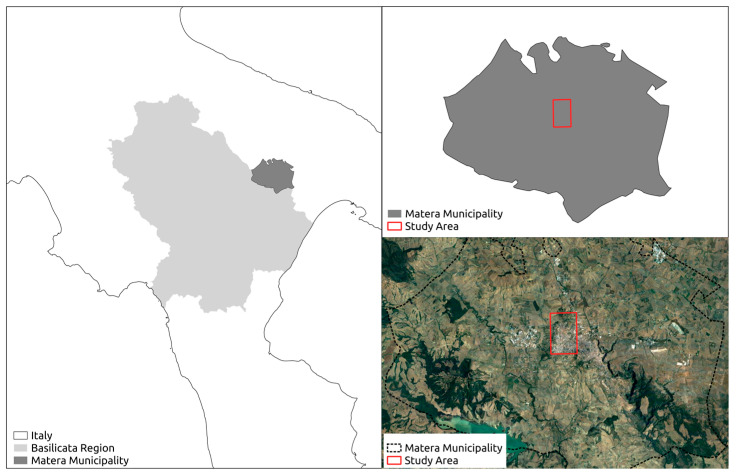
Location of the study area.

The proposed method was tested on an urban area of the municipality of Matera in the Basilicata region, Italy (Figure 1).

The municipality of Matera covers a surface area of 392.08 square kilometers and is characterized by a population of 59,748 inhabitants. Its population density is equal to 152.39 inhabitants/square kilometers. The municipality of Matera is internationally recognized as the “City of Sassi” since it is characterized by prehistoric caves carved into the rock, which represent some of the first human settlements in Italy. In 1993, Matera’s Sassi was the first site in Southern Italy recognized as a World Heritage Site by UNESCO, called a “Cultural Landscape”. Matera was also recognized as the European Capital of Culture in 2019. These events increased its popularity over time at the national and international levels.

Over the years, except for the tourism sector, the city’s economy has deteriorated. According to the national population census [56], the population of Matera decreased over time, while soil consumption increased [57], resulting in a progressive depletion of natural resources. In fact, even if Matera is characterized mostly by rural areas, its urban areas have expanded. For this reason, the municipality of Matera is considered a useful case study to analyze how the changes in urban development and vegetation cover happened during the time. The aspects related to the transformations of the territory, together with the historical interest and the size of the inhabited center, led the authors to consider Matera as a significant case study that could lead to highly transferable results.

Specifically, the proposed method was tested on an urban area in the north-west part of the municipality of Matera, which is represented in red (Figure 1). This is an expansion area that covers a surface of 6.59 square kilometers, which represents 1.70% of the whole municipality. Over the years, it has been characterized by an expansion of the built-up area due to the construction of new residences, urban services and commercial/productive blocks. These key factors contributed to the steady increase in land consumption and, therefore, the proposed spatial-temporal analysis can provide a better understanding of how urban planning strategies and policies affected the land cover changes.

### 2.2. Remote Sensing Imagery Dataset

The training and validation tasks of the U-Net model are developed using the “Semantic Segmentation of Aerial Imagery” dataset. For the model training and validation, the selected samples are 65 images and 7 images, respectively. To overcome the limitation of the small image sample size, the authors adopted several data augmentation techniques to improve the sufficiency and diversity of the training and validation data, generating a synthetic dataset [58]. The data augmentation operations consist of an under-sampling technique in which an image is randomly cropped multiple times, at a crop size of 512 × 512. The robustness of the model is enhanced by randomly flipping, rotating, translating, side viewing and zooming input images with the respective masks. In addition, all inputs are adjusted; setting their saturation, contrast and brightness; converting the color space and band order; and adding Gaussian noise and filtering operations randomly [59]. According to the data augmentation strategy adopted, the images are not treated with the same data augmentation operations. Given that the maximum number of images employed for training and validation tasks are equal to 715 and 77, respectively. 

**Figure 2:**
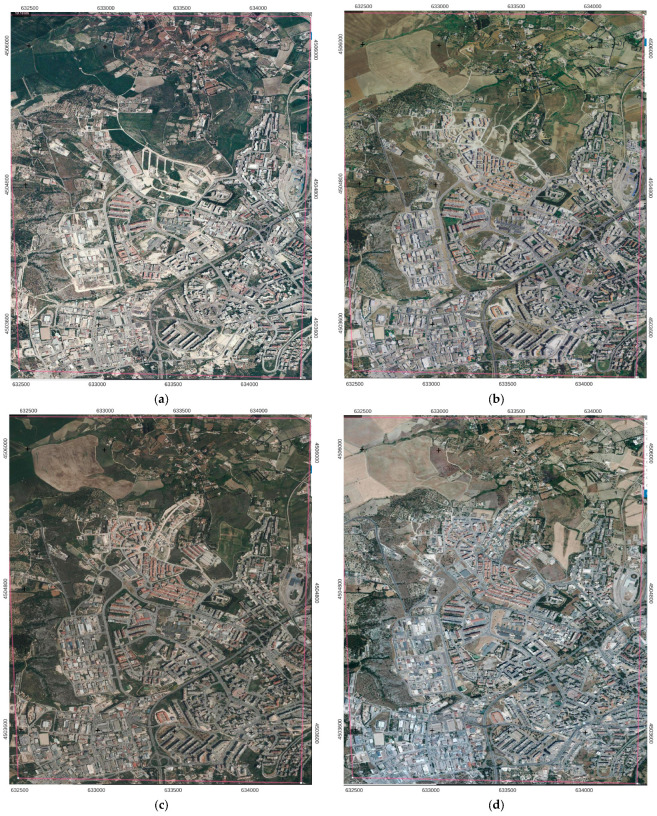

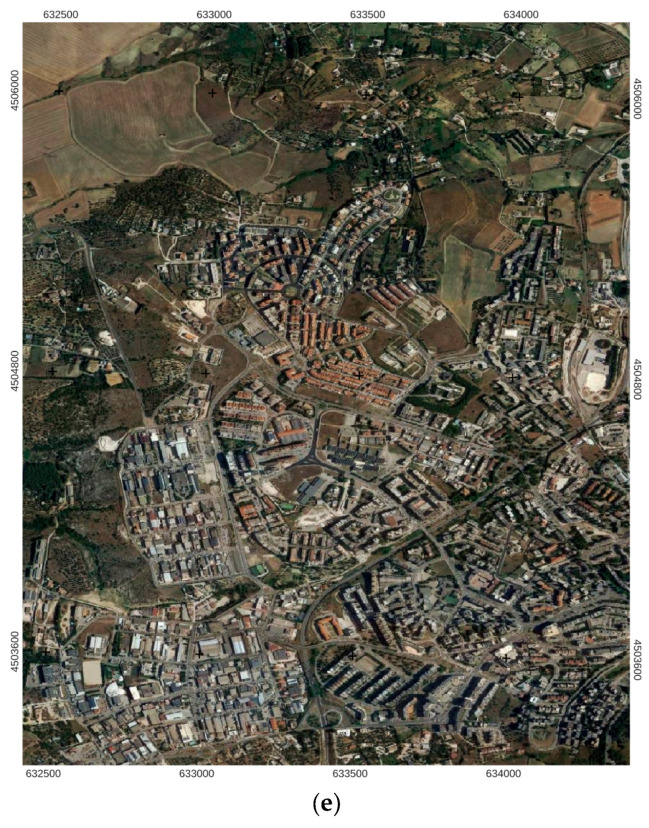
Testing dataset: (**a**) 2000; (**b**) 2006; (**c**) 2011; (**d**) 2017; (**e**) 2020.

To determine the built-up and the vegetation modifications for the 2000–2020 period, the trained and validated U-Net model was applied to 2000, 2006, 2011, 2017 and 2020 satellite images of the study area [60]. In particular, the 0.423 m pixel (approximately 1-foot) resolution orthoimages were considered (Figure 2).

To test the accuracy of the model, the authors compared the built-up and the vegetation polygons predicted through the application of the trained and validated U-Net model to the natural color orthoimages covering the case study area with those retrieved from the National Synthesis Database (DBSN) [61]. DBSN has a geotopographical database structure for medium-scale representations and represents the ground truth dataset. 

## 3. Methodology

The analysis of urban and greening development over time was developed by quantifying the amount and by evaluating the distribution of the built-up and vegetation cover at different periods, combining innovative RS technologies and GIS potentialities. The RS analysis of time series aerial imagery was performed using the U-Net model. 

The trained and validated U-Net model was tested through the classification and segmentation of buildings and vegetation areas from the natural color orthoimage of a specific urban context. For this study, only the buildings and the vegetation classes were considered. 

The authors imported and vectorized the output of the tested model on a GIS platform, which, in this study, was the open source QGIS Desktop software [62]. 

To assess the accuracy of the classified and segmented results, the authors compared the building and vegetation polygons with those derived from DBSN. The authors quantitatively assessed the accuracy of the model to predict the buildings and vegetation polygons by determining the F1-Score. 

Once the accuracy of the U-Net model was assessed, in order to determine changes in the vegetation cover and built-up area, the authors evaluate specific built-up and vegetation density indicators. In particular, the output of the built-up area and vegetation cover was imported in QGIS and processed to quantitatively evaluate the changes in the vegetation and urban features in space and time. 

**Figure 3:**
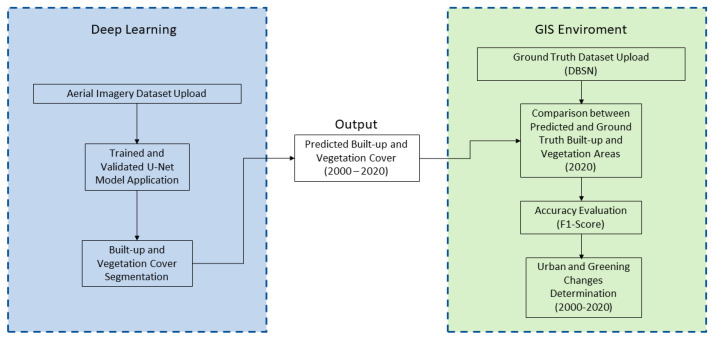
Methodology workflow.

Figure 3 schematically shows the methodology workflow.

### 3.1. Urban and Greening Classification and Segmentation

#### 3.1.1. Training and Validation of the U-Net Model

In this research, a U-Net model with a RELU activation function was used. The loss function considered was the IoU-based loss function, also known as the Jaccard index. IoU is a standard region-based performance metric for image segmentation problems. Generally, IoU is adopted to measure the similarity and calculates the ratio of the intersection and the union between the prediction and the ground-truth object in an image (Equation (1)). It is characterized by the properties of scale invariance, symmetricity, and nonnegativity. Its value ranges from 0 to 1, where 0 represents no similarity and 1 is a perfect equivalence between the predictions and the ground truth labels. The loss function considered incorporates the IoU measure following Equation (2).

(1) $$\mathrm{IoU}=\frac{\mathrm{TP}}{\mathrm{FP}+\mathrm{FN}+\mathrm{TP}}$$

(2) IoU−based Loss=1−IoU

In Equation (1) TP represents true positives, which is the number of correctly predicted elements; FP is false positives, which is the number of entities incorrectly identified as elements to be predicted; FN is false negatives, which represent the elements to be predicted incorrectly classified as elements of another category.

Adam was used as the model optimizer [63] with an initial learning rate of 0.001 and default hyper-parameters β1 = 0.9 and β2 = 0.999. The validation task was carried out during training and its loss was calculated and monitored. If validation loss did not improve within four epochs, the learning rate was reduced by a factor of 0.5. To prevent overfitting, the training was stopped if the validation loss did not improve within 100 epochs. At the end of the training process, the model associated with the lowest validation loss was saved. The training and validation tasks are carried out on Google Colab Pro Plus Environment, using 52 GB of RAM and the GPU NVIDIA Tesla P100. 

All the projects were implemented in Python and the training was developed using Pytorch with a TensorFlow backend as the deep learning framework. Supplementary Materials (https://github.com/ingegnerevitale/U-Net-Semantic-Segmentation-Aerial-Images accessed on 5 March 2023).

Precision, recall and F1-score [64] were selected to measure the performance of the U-Net model (Equations (3)–(5)).

(3) $$\mathrm{Precision}=\frac{\mathrm{TP}}{\mathrm{FP}+\mathrm{TP}}$$

(4) $$\mathrm{Recall}=\frac{\mathrm{TP}}{\mathrm{FP}+\mathrm{FN}}$$

(5) $$\mathrm{F1-Score}=\frac{2\cdot \mathrm{Precision}\cdot \mathrm{Recall}}{\mathrm{Precision}+\mathrm{Recall}}$$

#### 3.1.2. Testing the Accuracy Assessment of the Model

The U-Net model was applied to the case study, generating the predicted masks of the built-up and the vegetation polygons. These masks were imported into the open source QGIS Desktop software, converted into vectorial layers and compared with the label masks belonging to the ground truth dataset.

A comparison between the real and predicted built-up and vegetation polygons was needed to verify that the spatial autocorrelation between training and testing data was removed. As the training and validation tasks were developed using an imagery dataset of an urban environment considerably different from that of the case study in terms of spatial and formal characteristics [65], the testing accuracy removed the spatial autocorrelation between the real and predicted labels. 

The authors considered the F1-score (Equation (5)) as a performance measure of the model prediction accuracy, referring to a geographic area that the model had not experienced before. 

The accuracy of the results in terms of the built-up area and vegetation cover prediction was assessed using the 2020 orthoimage prediction mask, as this was the most recently analyzed official orthoimage of the study area. Once the accuracy of the model was defined, the urban and greening development change analysis of the 2000–2020 period was performed.

### 3.2. Urban and Greening Changes Analysis

To analyze the urban and greening changes in space and time, the output of the U-Net model for 2000, 2006, 2011, 2017 and 2020 years was processed according to defined urban and greening development indicators.

The most used indicator to analyze urban development—both compact and dispersed—was the population density, which was the number of inhabitants per spatial unit. However, this indicator did not allow us to assess how much built-up area was extant. The most relevant measure of density to assess the real development of urban areas is built-up area density. This measure puts pressure on greenspace conservation [66]. The most accurate measure to evaluate built-up density is the floor area ratio (FAR) [67]. This measure is commonly used to express the urban development intensity, limit the intensity of land use, lessen the environmental impacts of urban development and control the mass and the scale of urban development. FAR is typically calculated by dividing the gross floor area of a building by the total land area or the total buildable land area. Another measure of built-up density is the building coverage ratio (BCR), expressed as a ratio between the footprint area of a building and the total land area or the total buildable land area. The BCR measure ensures open spaces in the land, prevents overcrowded housing and secures neighborhood emergency evacuation measures. Since no information concerning the floor area value can be retrieved from the U-Net model’s automatic classification and segmentation of buildings and no data about the height of the buildings are available from 3D models, the authors utilized the BCR as the built-up area density measure.

The vegetation cover delivers different ecosystem services, such as climate regulation, air purification and runoff control, as well as contributing to enhancing human health and well-being [68]. Existing research has shown that urban development—both compact and dispersed—has a high influence on the quantity, connectivity, size, quality and accessibility of urban spaces [69,70,71]. Based on these observations and the available data, the authors evaluated the density of vegetation cover to measure its quantity and its distribution changes across space and time. The density of vegetation cover is calculated as the ratio between the vegetation cover predicted by the U-Net model and the reference area.

The indicators used for urban and greening development analysis in space and time are listed in Table 1.

## 4. Results

### 4.1. Accuracy Assessment of the U-Net Model

As stated in the methodological section, the U-Net model was trained and validated using the IoU-based loss function achieving precision, recall and F1-score values of 0.63, 0.84 and 0.72, respectively. The trained and validated U-Net model was then applied to the natural color orthoimages covering the study area for a period of 20 years, employing five different time points, namely 2000, 2006, 2011, 2017 and 2020. 

To assess the accuracy of the trained and validated U-Net model prediction, the real and predicted built-up area and vegetation cover were compared, using 2020 as the reference period. The overall accuracy of the U-Net model application was quantitatively assessed through the evaluation of the F1-score, considering the real and predicted built-up and vegetation polygons. 

According to the ground truth, in 2020, 40.37% of the overall study area was characterized by a built-up area, while 53.06% represented an area covered by vegetation.

The F1-score values show a great level of accuracy for both built-up areas and vegetation cover, reaching values of 0.95 and 0.97 (Table 2).

These results indicate a very good level of accuracy, considering that the U-Net model was applied to geographic areas it had never experienced before. This implies that the real and the predicted features are very close. 

**Figure 4:**
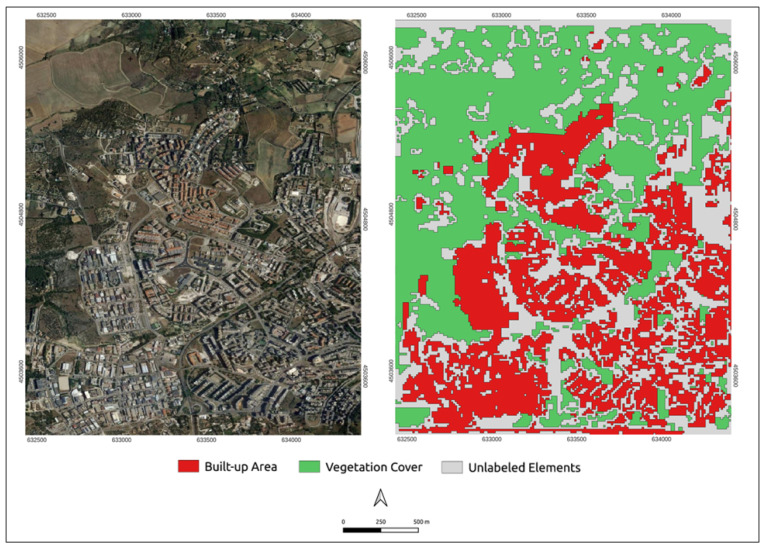
Predicted built-up area and vegetation cover for 2020.

From the built-up area and vegetation cover prediction (Figure 4), it can be seen that there are some built-up polygons (FP) in the peripheral areas and some voids (FN) within the denser urban environment, the expansion area and the industrial/productive zone that were not detected. Looking at the vegetation cover prediction, some predicted vegetation polygons that are non-vegetation can be found (FP). However, there are many real vegetation polygons that the model did not predict (FN). 

Once the accuracy of the model to predict the built-up and vegetation cover was assessed in reference to 2020, all the analyses concerning the built-up and vegetation changes for the 2000–2020 period were carried out using the predicted masks.

### 4.2. Urban and Greening Changes Analysis Results

To determine the built-up and vegetation modifications over the years within the study area, the U-Net model was applied to 2000, 2006, 2011, 2017 and 2020 orthoimages. 

For every orthoimage, the built-up and vegetation polygons were classified and segmented within short computational times to assess the changes in these urban elements over time.

**Figure 5:**
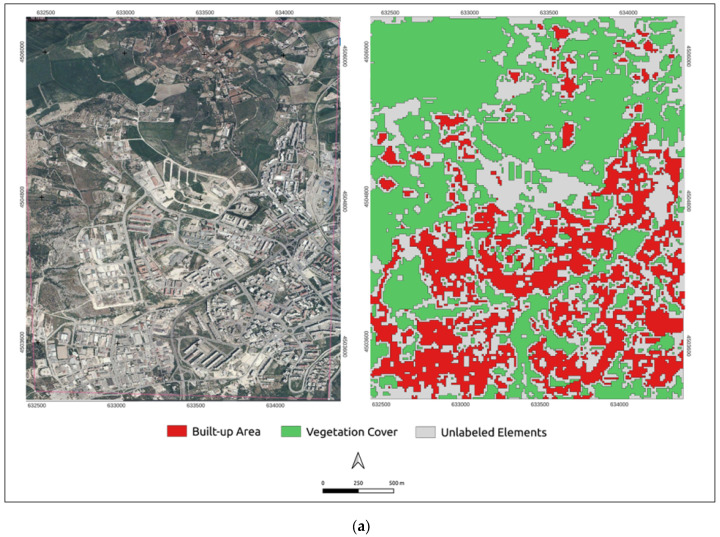

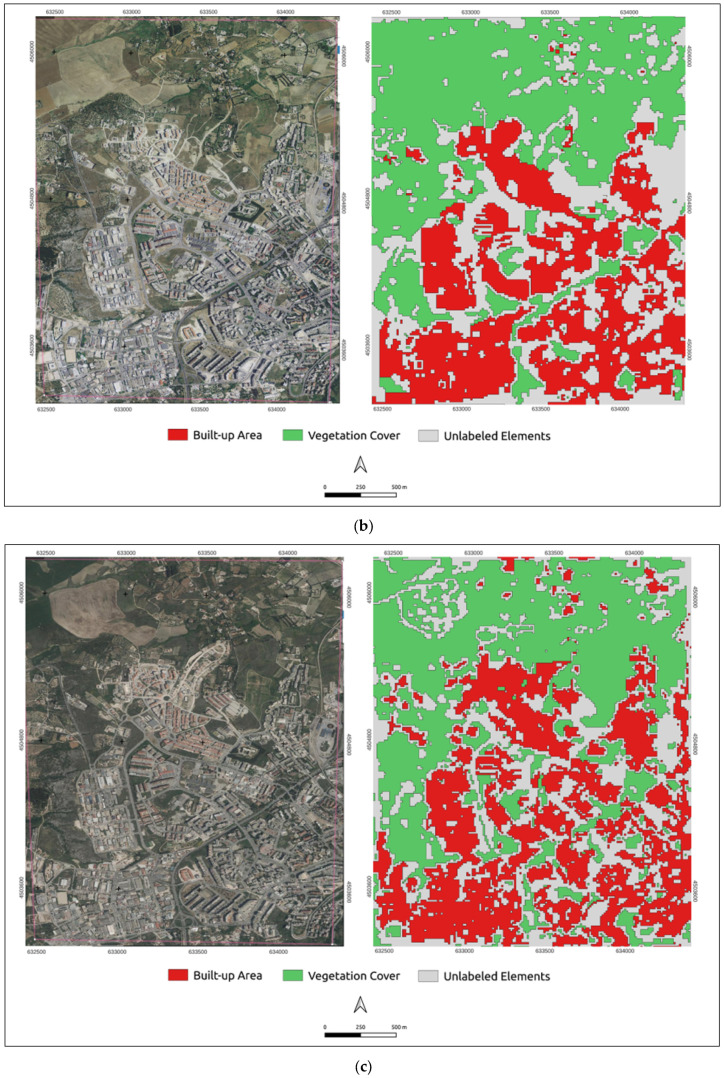

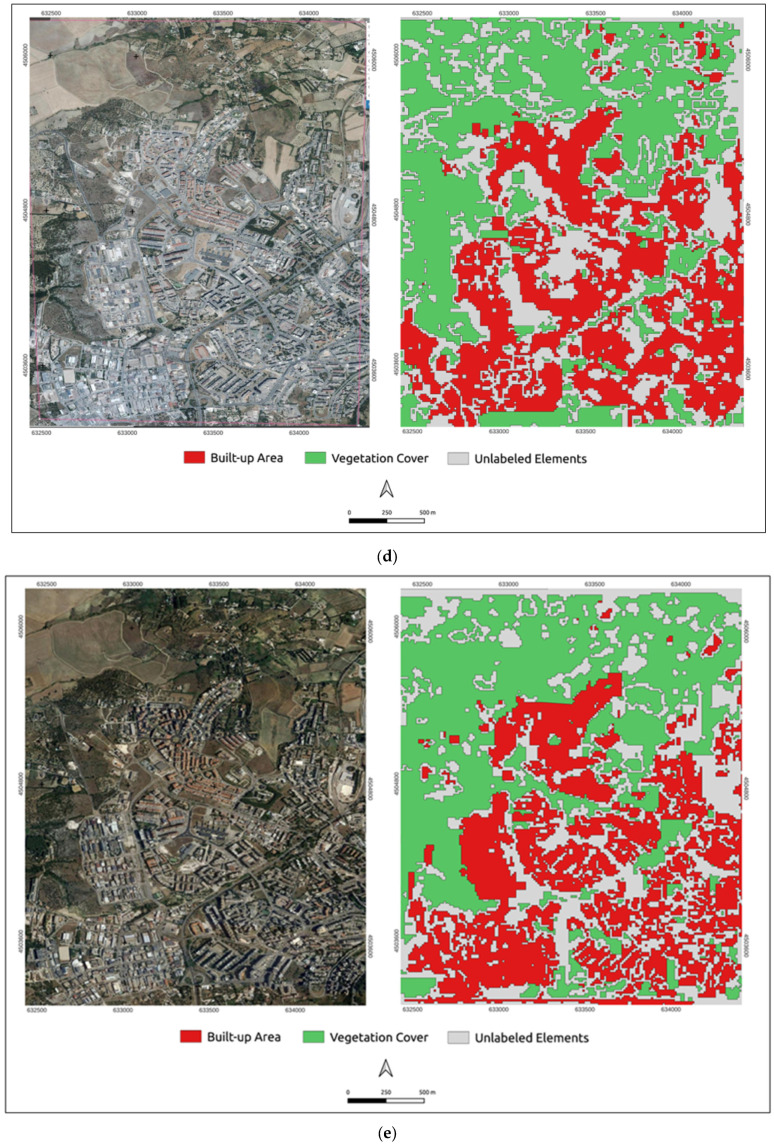
Predicted built-up area and vegetation cover from 2000 to 2020: (**a**) 2000; (**b**) 2006; (**c**) 2011; (**d**) 2017; (**e**) 2020.

In Figure 5, the predicted shapes of the built-up area and vegetation cover retrieved from the U-Net model application are presented, for all the investigated time points, in red and green, respectively. Since the U-Net model is trained and validated to predict only the built-up and vegetation polygons, the unclassified features are defined as unlabeled elements and are represented in gray. 

Figure 5 highlights a distinct change in the spatial patterns of the built-up area and vegetation cover between 2000 and 2020. 

After the application of the U-Net model to predict the built-up and vegetation cover for every year under investigation and the assessment of their aerial coverage, the indicators defined in the methodological section were assessed to analyze the urban and greening changes over time. 

The surface coverage in hectares and the density in percentages of built-up area, of vegetation cover and unlabeled elements for the five time points are summarized in Table 3.

The classification and segmentation of the natural color orthoimages from 2000 show that most of the study area is covered by vegetation and by unlabeled elements, representing 272.31 ha (41.33%) and 239.16 ha (36.30%), respectively, whereas the built-up area covered 147.4 ha with a density rate of 22.38%. 

Similarly, in 2006, the greatest share of the total study area was the vegetation cover at 253.64 ha (38.49%), followed by unlabeled element coverage at 218.71 ha (33.19%) and built-up area coverage at 186.56 ha (28.31%). 

The results of the classification for the 2011 orthoimage show that the highest density rate was related to the vegetation cover, reaching 246.82 ha (37.46%), while the built-up area and the unlabeled elements coverage were equal to 194.95 ha (29.51%) and 217.64 ha (33.03%), respectively.

Processing the 2017 aerial image, the results demonstrate that the unlabeled elements coverage was equal to 217.96 ha, with a density rate of 33.08%. The built-up area and vegetation cover, respectively, were 202.02 ha and 238.93 ha, which, in terms of density rate, is equal to 30.66% and 36.26%, respectively. 

From the application of the DL model to the 2020 orthoimage, it can be seen that the scenario is quite stable with respect to 2017. Indeed, built-up area coverage of 202.03 ha, with a density rate of 30.66% was obtained. The vegetation cover for 2020 was 238.49 ha (36.19%), while the unlabeled elements coverage was equal to 218.40 ha (33.15%).

The coverage and the density of built-up area, vegetation and unlabeled elements changes for the five periods (2000, 2006, 2011, 2017 and 2020) are shown in Table 4.

Considering the 20-year observation period, the greatest pattern changes in the urban and greening development for the case study occurred between 2000 and 2006. During this period, the built-up area increased by 39.12 ha, with a density increase of 5.94%, compared to the previous amount of cover. In contrast, vegetation cover and unlabeled elements density rate were reduced by 2.83% and 3.10%, resulting in a surface loss of 18.67 ha and 20.45 ha, respectively. 

From 2006 to 2011, the built-up area continued to increase by 7.89 ha with a density rate of 1.20%, while the vegetation cover surface decreased by a value of 6.82 ha (1.04%). The area of unlabeled elements declined by 1.07 ha, with a density rate reduction of 0.16%.

During the 2011–2017 period, the built-up area density continued to increase with a density rate of 1.15%, around 7.56 ha, while the vegetation cover density continued to decrease with a density rate of 1.20%, around 7.89 ha. The density rate of unlabeled elements slightly increased (0.05%), resulting in an increased area equal to 0.32 ha. 

During 2017–2020, the surface area and, thus, the density rate of the built-up area did not vary. The density rate of the vegetation cover and the other elements decreased and increased, respectively, by 0.07%, causing a loss and an increase of 0.44 ha and 0.43 ha.

Generally, taking into consideration the overall study period (2000–2020), the built-up area showed a remarkable area increase of 54.58 ha, which equaled a density increase of 8.29%. In contrast, the vegetation cover diminished by 33.82 ha, with a rate density reduction of 5.14% in the same period. From 2000 to 2020, the coverage area of predicted unlabeled elements decreased by 20.77 ha, for a density rate equal to 3.15%. 

Generally, the results demonstrate a series of urban and greening changes in the study area for the analyzed period (2000–2020), according to which, the study area experienced significant built-up area expansion, causing relevant vegetation cover decline. This urban and greening trend is found to be consistent with the results of a different study [57].

## 5. Discussion

The obtained results indicate that significant urban and greening cover changes occurred from 2000 to 2020. In fact, in the overall study area, during the 20-year analyzed period, the built-up area density increased by 8.28%, while the vegetation cover density decreased by 5.13%. This research finding indicates that the study area experienced a growing urbanization phenomenon, which led to a decline in vegetation cover. 

Specifically, the continuous urban development and the decrease in vegetation cover shown by the urban and greening changes analysis of the period between 2000 and 2020 are related to the realization of new residential areas and urban services in the northern part of the study area and to the completion of industrial/productive areas in the western part. The construction of new residential buildings and the consequent realization of urban services during this period followed an urban planning policy combining the core part of the urban area and the peri-urban area. The completion of the industrial/productive area, on the other hand, took place in the location of already-established enterprises. This land cover development pattern may be determined by population growth, which has led to a demand for new residential areas and new urban services as well as new job opportunities. 

The significant development pattern in the north-west side of the study area occurred between 2000 and 2006 when new residences were built in the north of the study area and new industrial/productive buildings were built in the west. In particular, the new residential zone was connected to the main urban zone, bringing together the core part of the urban area and the peripheral area. The U-Net model, through the classification and segmentation of the 2000 and 2006 natural orthoimages, predicts this development well. The built-up expansion trend and the reduction of the vegetation cover are also confirmed by the numerical results obtained from the application of the urban and greening indicators in the 2000 and 2006 prediction masks.

From 2006 to 2011, the expansion of the residences in the northern part of the study area continued through the realization of a new transitional zone between the urban and peripheral sides, resulting in a loss in vegetation cover. The cause of this residential expansion was a new important street connection. Moreover, during this period, the expansion activity also involved the growth of the industrial/productive zones in the west of the study area. The trend of urban development and greening loss is confirmed by the proposed and applied methodology. 

In the 2011–2017 period, the residences connecting the core part of the study area to the peripheral areas and the industrial/productive zone in the north and in the west, respectively, were almost completed, causing the complete removal of the vegetation cover in those areas. The applied DL method predicts these changes well. 

Between 2017–2020, no significant change occurred. During this period, some buildings in the north part of the study were constructed in the already built-up area, resulting in no further land consumption. This is confirmed by the model prediction.

Comparing the obtained results in terms of prediction with the changes shown by the orthoimages, the trend identified by the application of the proposed methodology is close to the trend shown by the analyzed orthoimages. Furthermore, the numerical results obtained from the application of the proposed indicators confirm the development of the built-up area and the decreasing vegetation areas, as assessed by [57].

Looking at the obtained geography of urban and greening changes for the study area, a phenomenon of peripheral urban development can be observed. These results suggest that decentralized planning policy affected this area, resulting in a sprawl of urban development. This analysis also shows how the loss of vegetation cover outside the core part of the urban area is not compensated for with the addition of new green spaces.

As demonstrated by the results, even if these kinds of planning policies promote the efficient integration between core and peripheral areas thanks to the construction of integrated urban and territorial services, such as transportation systems and high-level services, it results in a depletion of vegetation cover, causing a loss of ecosystem services. The higher the rate of urban development outside the inner city, the higher the natural resources and vegetation consumption. 

The results obtained from urban and greening development changes during the 20 years from 2000 to 2020 show that the DL model applied for the classification and segmentation of the built-up and vegetation cover predicts, with short computational times, these urban features with a very good level of accuracy. The described results show, in fact, the adequate capacity of the proposed methodology to very quickly identify urban and greening development changes over time from simple orthoimages related to different years.

Reaching an F1-Score of 0.95 and 0.97 for the built-up area and vegetation cover, respectively, the demonstrated accuracy of the proposed U-Net model prediction on a geographic area that the model had not experienced before was very good. This means that the spatial autocorrelation between the real and the predicted labels is almost completely removed.

The proposed methodology, based on the integration between the DL technology and GIS techniques, can be employed as a rapid system to estimate the built-up area and the vegetation cover with a good level of accuracy, providing a quantitative assessment of the urban and greening development changes over time. As the proposed integrated methodology represents an appreciable indicator of urban and greening growth and a predictor of future urban and greening development directions, it can be used by planners, decision makers and stakeholders to monitor and predict land cover changes in urban and territorial areas. 

The proposed urban and greening change detection methodology, combining the RS and GIS potentialities, delivers useful information about land use dynamics patterns, helping planners and decision makers to realize sustainable land management. It can be used as a rapid tool to quantify and visualize the real consumption of natural resources, such as vegetation and soil, in a semi-automatic way. Furthermore, it can also be used as an early warning system to identify land cover changes occurring without authorization or permission. 

In this study, the proposed tool was tested on a pilot area in the municipality of Matera. The obtained results show that this tool can be applied to other areas, which can be bigger than the one analyzed here, in order to assess the urban and greening changes occurring over time as well as to predict the future urban and greening development trajectories in larger urban and territorial contexts.

The main challenge of the proposed research is to compare the orthoimages related to different periods. Indeed, the different exposures of the aerial images determined using new and advanced cameras over time cannot have similar results in terms of built-up and vegetation cover prediction. Additionally, weather conditions have a significant influence on orthoimage processing. These different orthoimages conditions affect the obtained results. For example, the classified and segmented built-up coverage from the aerial image from 2000 is slightly underestimated in comparison to the real situation due to the clearness of the orthoimage, which does not make it possible to distinguish the color differences between the buildings and the unlabeled elements. On the contrary, the predicted built-up areas for 2006 seem to be good, as it is characterized by an identifiable color. Looking at the classified and segmented vegetation cover from the 2011 orthoimage, the vivid green color of the vegetation facilitates the identification of this category by the DL model. The green color of the vegetation areas within the 2017 orthoimage, on the other hand, is not so vivid and, therefore, the vegetation cover is underestimated by the U-Net model. 

Despite this, the trained and validated U-Net model predicted the built-up area and vegetation polygons very well and the proposed methodology provides a good estimation of the built-up area and vegetation development over the analyzed period. The limit of the different orthoimages, therefore, became an opportunity to test the ability of the proposed methodology to predict the built-up area and vegetation cover and assess the urban and greening development changes with a good level of accuracy in a very rapid way. Having a homogeneous, updated and open database of natural orthoimages would help to overcome this limitation. 

The proposed research work may be improved by testing other DL architectures that are more accurate to achieve better performances in building and vegetation cover prediction. Other labels, such as bare soil, roads and the differences between urban and rural green areas, may be added to comprehensively assess LULC changes over time, focusing on different urban features. Moreover, the proposed analysis based on the integration between DL and GIS technologies may be deepened by introducing socioeconomic and environmental indicators to analyze the drivers and the impacts of urban and greening development over time and space.

## 6. Conclusions

In this paper, the authors proposed a combined method based on the application of a DL model to aerial imagery in order to assess the urban and greening changes over time through a spatial analysis of the built-up area and vegetation cover. Specifically, the authors used a U-Net model with a RELU activation function that was trained and validated using the “Semantic Segmentation of Aerial Imagery”. The U-Net model was tested by classifying and segmenting the built-up area and the vegetation cover of an urban area of the municipality of Matera in the Basilicata region (Italy). The predicted built-up area and vegetation polygons were then uploaded to the GIS environment to assess, through specific density indicators, the land cover changes over a period of 20 years from 2000 to 2020. 

The accuracy assessment of the built-up and vegetation polygons prediction for 2020 resulted in F1-score values of 0.95 and 0.97, respectively. This result quantitatively demonstrated the goodness of the applied DL model to predict these two urban features. The qualitative comparison between the orthoimages and the prediction of the built-up area and vegetation cover for the orthoimages from 2000, 2006, 2011 and 2017 confirmed this ability of the U-Net model. 

The geospatial analysis aimed at identifying urban and greening development in the study area showed that during the analyzed period (2000–2020) the study area experienced a remarkable increase in built-up density (8.28%) and a significant reduction in vegetation cover density (5.13%). Moreover, looking at the geography of these urban and greening changes, a peripheral urban development can be observed. This identified trend in the loss of vegetation cover shows, therefore, how the urban development outside the core area of the denser urban environment puts pressure on vegetation cover, resulting in negative impacts on the livability and sustainability of urban and territorial areas. 

The proposed method demonstrates how the assessment of land use through the application of innovative DL models and geospatial analysis techniques is very effective for assessing and monitoring the net effect of urban planning policies in terms of land cover changes. Thanks to the proposed methodology, in fact, a rapid estimation of urban and greening changes over time, with a very good level of accuracy, can be obtained. It can also be a useful support system for urban planning practitioners in order to find suitable strategies to complement urban development with the delivery of the crucial ecosystem services of the natural environment.

## Figures and Tables

**Table 1:** Urban and greening development indicators.

Indicator	Variable
Built-Up Area Density	Area of Built-Up to Reference Area
Vegetation Cover Density	Area of Vegetation Cover to Reference Area

**Table 2:** Accuracy assessment of the U-Net model for the built-up area and vegetation cover prediction.

Labels	F1-Score
Built-Up Area	0.95
Vegetation Cover	0.97

**Table 3:** Coverage and density of urban and greening in 2000, 2006, 2011, 2017 and 2020.

Labels	2000		2006		2011		2017		2020	
	Coverage (ha)	Density (%)	Coverage (ha)	Density (%)	Coverage (ha)	Density (%)	Coverage (ha)	Density (%)	Coverage (ha)	Density (%)
Built-up Area	147.44	22.38	186.56	28.31	194.45	29.51	202.02	30.66	202.03	30.66
Vegetation Cover	272.31	41.33	253.64	38.49	246.82	37.46	238.93	36.26	238.49	36.19
Unlabeled Elements	239.16	36.30	218.71	33.19	217.64	33.03	217.96	33.08	218.40	33.15
Total	658.91	100	658.91	100	658.91	100	658.91	100	658.91	100

**Table 4:** Coverage and density of urban and greening development between 2000–2006, 2006–2011, 2011–2017 and 2017–2020.

Labels	2000–2006		2006–2011		2011–2017		2017–2020		
	Coverage (ha)	Density (%)	Coverage (ha)	Density (%)	Coverage (ha)	Density (%)	Coverage (ha)	Density (%)	
Built-up Area	39.12	5.94	7.89	1.20	7.56	1.15	0.01	0.00	
Vegetation Cover	−18.67	−2.83	−6.82	−1.04	−7.89	−1.20	−0.44	−0.07	
Unlabeled Elements	−20.45	−3.10	−1.07	−0.16	0.32	0.05	0.43	0.07	

## Data Availability

The “Semantic segmentation dataset” employed in training and validation tasks is available at https://humansintheloop.org/semantic-segmentation-dataset/ (accessed on 23 January 2023).

## Supplementary Materials

Python code, input image data, and output predicted built-up area and vegetation cover masks can be accessed at: https://github.com/ingegnerevitale/U-Net-Semantic-Segmentation-Aerial-Images (accessed on 5 March 2023).
